# Evaluation of the Fetal Left Ventricular Myocardial Performance Index (MPI) by Using an Automated Measurement of Doppler Signals in Normal Pregnancies

**DOI:** 10.3390/diagnostics11020358

**Published:** 2021-02-20

**Authors:** Su-Min Kim, Soo-Young Ye

**Affiliations:** 1 Department of Obstetrics, Busan Well-High Woman’s Hospital, 95 Myeongji Ocean City 4-ro, Gangseo-gu, Busan 46764, Korea; tnals7614@gmail.com; 2 Department of Radiological Science, Graduate School, Catholic University of Pusan, 57 Oryun-daero, Geumjeong-gu, Busan 46252, Korea

**Keywords:** fetal ultrasound, fetal myocardial performance index (MPI), Tei index, K-index, cardiac interval, automatic detection

## Abstract

The myocardial performance index is widely used as an indicator of the heart’s performance. However, due to the subjective nature of ultrasonic testing, there are differences in the measurements among inspectors, requiring a quantitative and objective assessment. In this study, an automated program was developed to quantitatively evaluate the myocardial performance index (MPI) and the cardiac time intervals in the left ventricle for each trimester. One hundred and thirty-three pregnant women who visited the hospital for prenatal examinations were studied, and skilled inspectors obtained left ventricular blood flow waveforms from 47 fetuses in the 12 weeks, 54 fetuses in the 22 weeks, and 32 fetuses in the 31 weeks of pregnancy using a pulse Doppler mode of ultrasound equipment. The acquired images automatically measured the isovolumetric contraction time (IVCT), isovolumetric relaxation time (IVRT,) ejection time (ET), and filling time (FT), and calculated the Tei index (TI) and the K-index (KI); each interval was manually measured during the actual inspection for comparison. In this study, the ultrasonic Doppler waveform was objectively analyzed and measured by the automated program, and it will help with the evaluation of fetal heart function.

## 1. Introduction

The classical indicators for evaluating the function of the left ventricle in an echocardiogram show that the heart is shaped like a sphere, causing many errors. To counteract this, the myocardial performance index (MPI), designed by Tei et al. [1], was created on the basis of the fact that the isovolumetric contraction time (IVCT) is increased in the event of systolic failure, and that the isovolumetric relaxation time (IVRT) is increased in the event of systolic and relaxation disorders. The MPI, also called the Tei index (TI), is produced by dividing the sum of the IVCT and the IVRT by the ejection time (ET), which can easily quantitatively evaluate the overall cardiac function by combining the heart systolic and relaxation functions and does not rely on geometric assumptions. If the heart function is abnormal, the isovolumetric time is extended and the ET is reduced, which in turn increases the MPI [1,2].

The MPI, meanwhile, measures each waveform using Doppler signals from the aortic valve and mitral valve inflow blood flow in the left ventricle, which is not measured in the same heart cycle, and thus it is difficult to determine if there is an excessive variation of heart rate. Friedman et al. devised a modified MPI measurement method for applying MPI to fetuses, which allows the Doppler measurement position to be obtained simultaneously from one cross-section by placing the position of the Doppler measurement tool in the middle of the ventricle inlet and outlet [3]. Hernandez et al. enhanced the reproducibility of measurements by using valve signals that appear as high signals in the waveform as valve clicks, on the basis of Friedman’s method’s high variability of measurements due to the absence of a clear reference point at the measurement site [4]. This modified myocardial performance index (Mod-MPI) has been verified by several researchers for clinical validity and is widely used to assess the function of the heart in intrauterine growth retardation (IUGR), preeclampsia, gestational diabetes, and twin-to-twin transfusion syndrome (TTTS) [5,6,7,8]. To this day, many authors have published research results on fetal MPI, but each study has shown a large difference in the results [8,9]. In general, the MPI is measured 0.53 ± 0.31 from mid-term to early term pregnancy (18 to 31 weeks pregnant) [3], but there has been no report of the standard time to do so. This is due to the nature of ultrasound testing, which must be measured at the subjective judgment of the examiner, and to overcome these problems, several recent studies have developed various methods of automatically measuring the MPI [10,11,12].

It is very important to use the left ventricular filling time to assess the performance of the heart. On the basis of this, Ahsan et al. developed a new index to measure the MPI and called it the K-index (KI) [12]. Since the KI is produced by dividing the sum of the IVRT and the IVCT by the ventricular filling time (FT) and is very closely related to an increase in placental blood flow, it is clinically more useful to assess the MPI on the basis of the filling time (FT) than using the ET.

Ultrasound examination has the advantage of being able to acquire images in real time using non-invasive testing methods, but it also has the disadvantage of showing differences in diagnostic results depending on the equipment and the inspector’s proficiency and knowledge. Because ultrasound is an operator dependent technique, objective and quantitative image evaluation is required.

In this study, we developed an automated program to calculate the MPI to measure the cardiac time intervals and TI and KI in the left ventricle of fetuses during pregnancy. In addition, we aimed to confirm the agreement between the values measured by automatic detection and the actual measurements taken during the test.

## 2. Materials and Methods

### 2.1. Subjects

This study is a prospective cross-sectional study conducted on 133 pregnant women who visited the hospital for prenatal examination from April 2019 to February 2020. The subjects were healthy single-fertile pregnant women without complications such as gestational diabetes and gestational hypertension, and were classified into 3 groups according to the gestational age: the early pregnancy women who visited at 12 weeks, the mid-pregnancy women who visited at 22 weeks, and the late pregnancy women who visited at 31 weeks. In addition, if a pregnant woman was in a high-risk group or an obstetrician had diagnosed IUGR during prenatal examinations, the subject was excluded from the study, as well as if the quality of the images was poor or if the ventricle inflow and outflow blood were not sufficiently included. Ultrasound examination was conducted by an ultrasound technician with more than 5 years of experience, and the Doppler waveform was obtained with minimal movement of the fetus. All tests were conducted under the mother’s voluntary participation and were approved by the Institutional Review Board (IRB) of the Catholic University of Busan (CUPIRB-2019-009) on 27 March 2019.

### 2.2. Data Acquisition

Doppler images of the left ventricle were obtained from pregnant women who had ultrasound tests performed during pregnancy. A total of 133 waveforms were analyzed, 47 in early, 54 in mid-, and 32 in late pregnancy.

**Figure 1:**
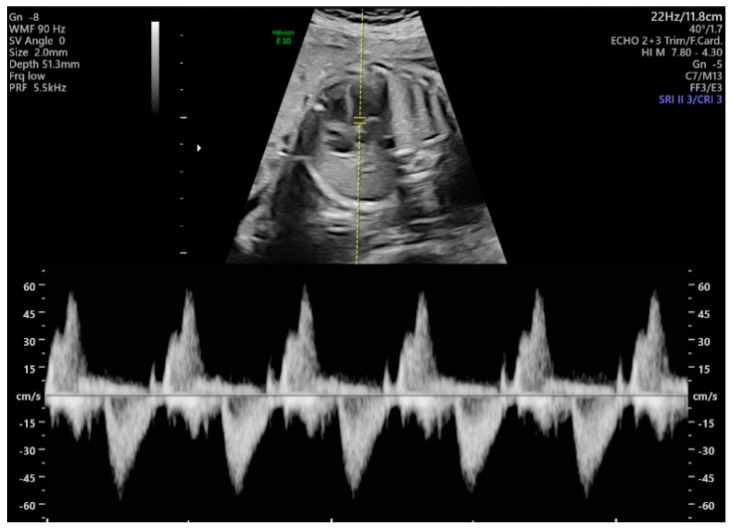
Two-dimensional (2D) images and Doppler waveforms of the measurements.

The methods used for acquiring Doppler waveforms for measuring the MPI were as follows. First, the aortic cortical part of the fetus was checked by turning the probe slightly in the direction of the fetus’ shoulder in the apical four-chamber view. Next, the pulse Doppler mode was executed, the sample volumes were positioned close to the aorta and the mitral valve, and the blood flow waveforms were obtained. Using the invert function during the examination, we set the left ventricle inflow blood flow to positive and the outflow blood flow to negative; the sweep speed was set to 5, and the sample volume size was fixed at 2 mm. Other ultrasound settings included low Doppler gain, 60 Hz wall motion filter, 8.3 kHz PRF (pulse repetition frequency), less than 0.4 thermal index and less than 0.7 mechanical index, and angle of insonation less than 20. The equipment used was a Voluson E10 ultrasound machine (GE Healthcare, Chicago, IL, USA) with a 3.5 MHz convex array probe (C2-D). The two-dimensional (2D) images and Doppler waveforms of the measurements are shown in Figure 1, including more than 4 cycles per fetus. During the examination, one operator performed a measurement of time intervals at the same time and stored both manual and original images. Automatic measurements were made on stored images. To assess intraoperator variability, we took measurements again by the same operator on a different time.

### 2.3. Signal Processing of Data for Automatic Measurment

First of all, the noise in the data was reduced for a smooth measurement of the time intervals due to the fact that the existing waveforms contained variation and discontinuity. The first Doppler waveform acquired was transformed into binary images, made into simple graph images along the contour, and low-pass filters were applied to eliminate the fine variation of the signal. The Butterworth low-pass filter (BLPF) was used in this study, which generates no ripple in the passband and attenuates unwanted frequency signals outside this band. The transfer function of the BLPF is

(1) $$\left|H\left(\omega \right)\right|=\frac{1}{\sqrt{1+{\left(\frac{\omega }{\omega_{0}}\right)}^{2n}}}\;\;$$

where ω is the frequency input and $\omega_{0}$ is the cut-off frequency. n means the order of the filter, and the higher the order, the higher the decrease rate increases, and the frequency at the point where $1/\sqrt{2}$ of the transmission function is located is the cut-off frequency. These operations can be obtained from Equations (2) and (3).

(2) $$\frac{1}{\sqrt{1+{\left(\frac{\omega_{-3dB}}{\omega_{0}}\right)}^{2n}}}=\frac{1}{\sqrt{2}}$$

(3) $$\omega_{-3dB}=\omega_{0}$$

After reducing the high-frequency signal of the graph using low-pass filters, we applied the peak detection function to find the regularity of the graph according to the heart cycle. Time when blood comes into the left ventricle consists of the early diastolic period (E wave) and the atrial contraction period (A wave). Unlike adults, fetuses are more dominant in A wave, and thus the most prominent value in the graph is A wave [13,14,15]. Therefore, in this study, we wanted to obtain each cardiac interval and the MPI on the basis of the maximum value of the A wave, and we used the automatic multiscale-based peak detection (AMPD) algorithm to automatically measure it.

AMPD is a peak detection method proposed by Scholkmann et al. [16] that calculates the local maxima scalogram (LMS) from the target signal and stochastically determines the location of the peak component. Finally, the peak position is detected using the fact that the standard deviation of the scalogram is minimal. AMPD has features that make it easy for users to detect peaks without having to enter specific variables. To detect peaks using AMPD, we first used the peak characteristics that rise in the positive direction. When the quantified Doppler signal with a total length of N is x, the three signals $\left\{x_{i-k},\;x_{i},x_{i+k}\right\}$ are compared with a sample clearance of k on both sides of $x_{i}$ to calculate the local maximum value of $m_{k,i}$.

(4) $$m_{k,i}=\{\begin{array}{cc} 0, & x_{i-1}>x_{i-k-1}\wedge x_{i-1}>x_{i+k-1}\\ r+\alpha , & otherwise \end{array}$$

If $x_{i}$ is the largest of the three compared values, the component value is 0, and any value r+α can be used to calculate the LMS matrix, where r is an arbitrary value between 0 and 1 and α is a constant 1. The LMS matrix contains information about the distribution of local maximum values.

(5) $$LMS\;Matrix=M=\left(\begin{array}{cccc} m_{1,1} & m_{1,2} & \ldots & m_{1,N}\\ m_{2,1} & m_{2,2} & \ldots & m_{2,N}\\ \vdots & \vdots & \ddots & \vdots \\ m_{L,1} & m_{L,2} & \ldots & m_{L,M} \end{array}\right)=\left(m_{k.i}\right)$$

The next step is to obtain the sum γ of each row of the LMS matrix. The total sum of each row in the γ array has a relatively small value.

(6) $$\gamma_{k}=\overset{N}{\underset{i=1}{\sum }}m_{k,i}\;\;,\;\;for\;k\in \left\{1,\;2,\ldots ,L\right\}$$

The vector γ contains the information about the scale-dependent distribution of the zeros. The global minimum of γ is called the “Lambda (λ) index” and is the largest local representation of the γ array in the size array. The λ value is used to reduce and relocate the previously obtained LMS matrix. The relocated LMS matrix can estimate the position of the peak by obtaining the standard deviation σ on the basis of the column.

(7) $$\sigma_{i}=\frac{1}{\lambda -1}\overset{\lambda }{\underset{k=1}{\sum }}{\left[{\left(m_{k,i}-\frac{1}{\lambda }\overset{\lambda }{\underset{k=1}{\sum }}m_{k,i}\right)}^{2}\right]}^{\frac{1}{2}},\;\;\;\;\;for\;i\in \left\{1,2,\ldots ,N\right\}$$

The peaks obtained using AMPD correspond to the A wave of the blood flow into the left ventricle and are the basis for each cycle on the graph. After setting the measurement criteria, we should determine the time of blood inflow and outflow through the process of local peak detection, which finds the maximum value by specifying a certain range. When a certain range is specified toward the negative direction of the A wave, 2 small peaks appear, corresponding to signals from the E wave and the aortic valve close to the A wave. The minimum value located between the 2 peaks is the starting point for the FT, which indicates the onset of the relaxation of the blood flow. By moving further to the left in the same way and specifying a certain range, the end point of the ET that corresponds to the maximum value of the (–) axis can be obtained. Then again, specifying a certain range on the right side of the A wave provides the FT’s end point and the ET’s starting point.

To assess intraoperator variability, measurements were taken again by the same method on a different cycle.

### 2.4. Calculating the MPI

**Figure 2:**
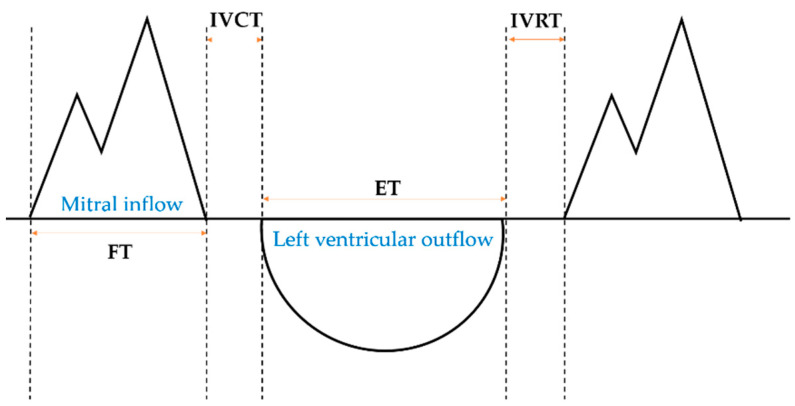
Schematic representation of the measurement of the cardiac time intervals. The horizontal axis of the graph represents time, and the vertical axis represents speed. FT, filling time; IVCT, isovolumetric contraction time; ET, ejection time; IVRT, isovolumic relaxation time.

The data obtained from the Doppler graph are signals for the beginning and end of the ET and of the FT, respectively, which can be used to obtain the ET, FT, IVCT, and IVRT and to calculate the MPI (Figure 2).

The MPI, which focuses on the contraction function of the left ventricle, is also called the Tei index (TI). It is expressed as follows [1]:

(8) $$TI=\frac{IVCT+IVRT}{ET}$$

KI is a type of MPI devised by Ahsan et al., focusing on the relaxation function of the left ventricle. It is expressed as follows [12]:

(9) $$KI=\frac{IVCT+IVRT}{FT}$$

**Figure 3:**
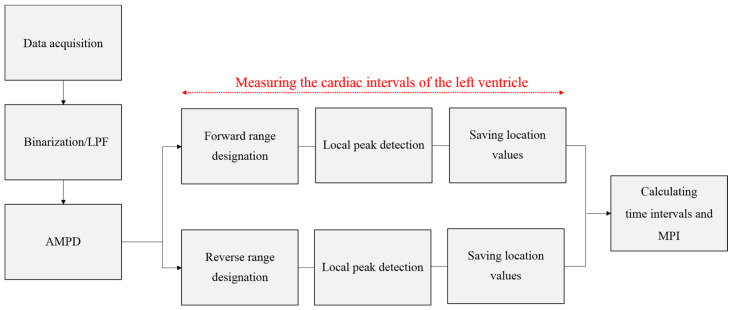
Flowchart of image processing conducted in this study. LPF, low-pass filter; AMPD, automatic multiscale-based peak detection; MPI, myocardial performance index.

The flowchart of the image processing conducted in this study is shown in Figure 3.

### 2.5. Statistical Analysis

The mean and variance of fetal cardiac intervals measured by automatic and manual method were compared. Intra-observer reproducibility was assessed using intraclass correlation coefficients (ICCs) with 95% confidence interval (CI). All statistical significance levels (*p*-values) in the data were tested below the 0.05 level and IBM SPSS Statistics version 25 (IBM Corp., Armonk, NY, USA) was used for the statistical analysis.

## 3. Results

### 3.1. Results of the Doppler Signal Processing

**Figure 4:**
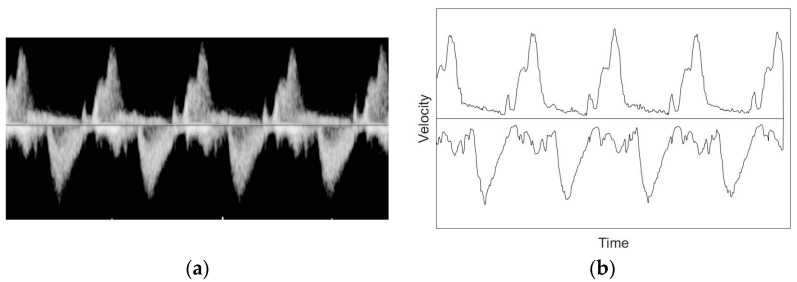
The original image acquired by the fetus in the early stages of pregnancy (**a**) and an image with binary and low-pass filters (**b**).

All images obtained through the examination were converted to simple graphs using BLPF for the ultrasound Doppler signals. Figure 4a is the original image acquired by the fetus in the early stages of pregnancy, and Figure 4b is the image with binary and low-pass filters. Most of the noise seen in the original image was removed, making peak detection easier.

**Figure 5:**
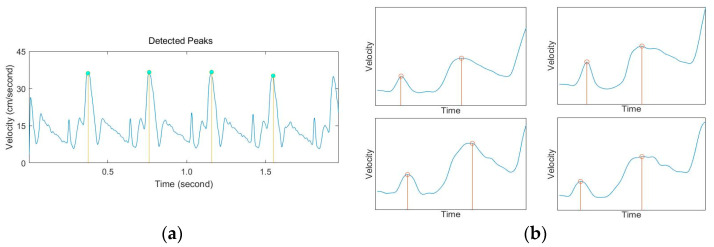
The result of performing the AMPD algorithm (**a**) and local peak detection in the Doppler waveform graphs (**b**).

Figure 5 shows the result of performing the AMPD algorithm and local peak detection in the Doppler waveform graphs. Four peaks, or four cycles, were found using AMPD, and the location information of each timing signal of a time interval could be detected through local peak detection.

**Figure 6:**
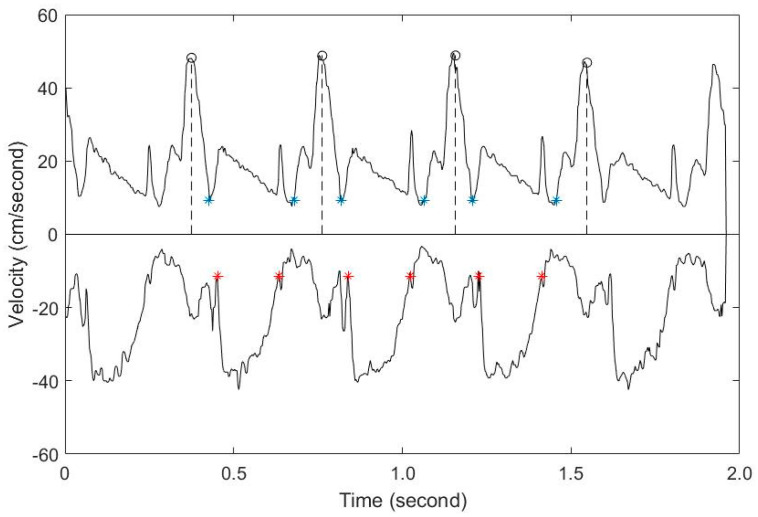
The results matched with the measured values.

After matching the previously obtained information with the original signal, we found the results of entering each time in the graph, which is shown in Figure 6. The blue-plotted part of the graph represents the blood flow of the left ventricular diastolic phase, and the red-plotted part represents the blood flow of the left ventricular systolic phase.

### 3.2. Results of the Measurments

In this study, data were obtained from 47 fetuses in the first trimester, 54 fetuses in the second trimester, and 32 fetuses in the third trimester, totaling 133 fetuses. The acquired data were used to detect the IVRT, IVCT, ET, and FT using an automatic program and to calculate the MPI. For comparison, each time interval and the MPI was measured directly in the same fetus during the examination as manual. The average value and standard deviation of each trimester were calculated, and the normality was demonstrated with the Shapiro–Wilk test. Since the *p*-values of all measurements, including the automatic and actual measurements in a whole gestational age, were higher than 0.05, the entirety of the measured data follows normal distribution. The maternal age, gravity of maternal, BMI (body mass index), and gestational age as subjects of study are shown in Table 1. The BMI was classified on the basis of weight gain during pregnancy through the guideline from the Institute of Medicine in 2009.

Table 2 shows intra-operator reproducibility of measurement using manual and automated methods. At all values, the intra-class coefficient of automatic measurements was shown to be higher than the manual measurements. All statistical significance levels (*p*-values) in the data were tested below the 0.05 level.

## 4. Discussion

The myocardial performance index (MPI) is useful for assessing fetal blood flow anomalies, due to its advantage of being able to assess both the relaxation and contraction performance of the left ventricle [1]. The MPI can be obtained simply by using a formula that combines the heart’s ejection and filling times, provided only the appropriate waveform is obtained for such measurements. Since 1995, when the MPI was first designed, conventional researchers have tried to use Doppler to measure the fetal MPI and to present criteria, but the results vary depending on the type of examination equipment, the quality of the images acquired, and the proficiency of the tester [8,9]. In particular, in the process of measuring the time interval directly on Doppler waveforms acquired during an examination, the location of the caliper specified by each inspector changes each time, which also causes variation in the measurement value. This is due to the limitation of ultrasonic testing being a semi-quantitative evaluation method, and thus it has been mentioned numerous times before that there is a need for quantitative and objective measurements. Therefore, in order to overcome the limitations of these ultrasound examinations, this study was conducted to develop algorithms applicable to actual clinical trials using automated programs. To obtain the MPI, ventricular ejection time (ET) and ventricular filling time (FT), as well as the isovolumetric contraction time (IVCT) and isovolumetric relaxation time (IVRT), should be measured first. In this study, the characteristics of the blood flow waveform were analyzed and the method of calculating the MPI after automatic measurement of time intervals at each cycle of the graph was applied. Therefore, automated measurements can be made in the same way in all cases, enabling objective measurements.

After Tei et al. first devised the MPI, Friedman et al. proposed a new method of simultaneously measuring both incoming and outgoing blood flow in a single test. Until now, this has been used primarily for the application of the MPI to fetuses, and the Doppler waveform was acquired in this study as well. In Friedman’s study, the MPI measured in 74 fetuses from 18 to 31 weeks of pregnancy was 0.53, and the MPI increased significantly as the number of weeks of pregnancy increased [3]. Hernandez et al., who measured the heart cycle on the basis of valve signals and who measured the MPI in 557 fetuses at 0.35 in mid-term pregnancy (19 weeks) and 0.37 in late-term pregnancy (39 weeks), similarly observed an increase in the MPI as pregnancy continued [4]. A number of subsequent studies have measured the MPI and have shown the same trend, but the measurements themselves vary considerably [3,4,8,9,11,17,18,19]. This is due to the characteristics of ultrasound testing, which require measurement with subjective judgment, and attempts to automatically measure the MPI using computers to overcome these problems were underway until recently. In Ahsan et al.’s case, the MPI was measured on the basis of the fetal electrocardiogram (ECG) signals during the test, and a total of 55 fetuses were obtained with a result value of 0.75, but there was no significant difference between the trimesters of pregnancy [12]. However, advanced studies have already shown that the MPI increases as the fetus grows, and it takes certain equipment and complex processes to extract the actual fetus’ ECG signals; thus, various inconveniences need to be dealt with prior to clinical applications. Later, Lee’s study developed a program to measure the TI on the basis of valve signals, and observed that the TI measured in 222 fetuses was 0.33 at the beginning of pregnancy, 0.50 at the end of pregnancy, and that the TI increased as the pregnancy continued [11].

Emphasizing the importance of the diastolic rather than the systolic phase, Ahsan et al. proposed a new MPI measurement method called the KI, and 55 fetuses were measured as 1.00 and 0.87 during and at the end of pregnancy, and the KI decreased as pregnancy continued. However, in this study, there was no significant difference in the KI in terms of the trimester of pregnancy in automatic measurement. It is theoretically correct for normal fetuses to increase in their KI as the IVCT increases by a certain amount as the number of weeks increases, but in Ahsan et al.’s study, as in this study, conflicting results were observed. In fetal circulation, some of the blood flow from the placenta goes directly through the foramen ovale to the left ventricle, but some reaches the left ventricle through the lungs, and the KI is assumed to be due to this complex flow of blood. Further research will be needed on whether the performance of a fetus’ heart affects the actual KI.

During pregnancy, cardiac time intervals and MPI change as the fetal heart grows and changes. The results of this study show that IVCT and IVRT increased as the number of weeks increased during pregnancy, and that the value of MPI increased as pregnancy continued. Such changes in each cycle over the duration of pregnancy can be explained as changes in the development of the fetal heart during pregnancy. As the fetus continues to conceive, the contraction ratio of the heart muscle increases and the thickness of the heart muscle increases, thereby increasing the fetal ventricular elasticity. This increase in relaxation ability causes E-wave to grow and consequently increases the value of MPI as pregnancy progresses.

In this study, the intra-observer reproducibility was confirmed in automatic and manual measurements. At all values, the intra class coefficient of automatic measurements was shown to be higher than the manual measurements. In other words, the automated program developed in this study indicates that fetal heart performance can be assessed quantitatively rather than manual method.

This study evaluated only healthy mothers and normal fetuses with no history of disease. This is the biggest limitation of this study, and the clinical validity of the automation program should be ensured later through comparative analysis with abnormal fetuses or high-risk mothers. The second biggest limitation in this study is the amount of data. It is difficult to represent the entire pregnant population because only 133 pregnant women were studied. However, the results of previously published prior studies differed from study to study due to the method of measurement, which relied on the subjective judgment of the examiner. This study is meaningful in that the self-developed automation program presented normal reference values of the fetal left ventricular MPI accordingly. Through this study, it is judged that it can be usefully applied as basic data for the quantitative evaluation of ultrasound examinations, as well as for the evaluation of fetuses’ heart functions in the future.

## 5. Conclusions

In order to quantitatively and objectively measure the left ventricular myocardial performance index of fetuses using ultrasonic Doppler, we developed an automated program to detect the IVCT, IVRT, ET, and FT and to calculate the MPI (both the TI and the KI). All processes, except data collection, were automated and compared with the actual measurements during the examination of the same fetus to determine the clinical usefulness of each measurement. The intra-observer reproducibility was confirmed in automatic and manual measurements, and all values using the automation program were higher than manual measurements. It is expected that the automated measurement program implemented in this study can be used effectively in clinical applications, and that it will help with the evaluation of fetal heart function such as IUGR, preeclampsia, TTTS, and fetal anemia.

## Figures and Tables

**Table 1:** General characteristics of subjects.

Variable		Number (%)
Maternal age (years)		
	<30	25 (18.80)
	30–34	54 (40.60)
	35–39	48 (36.09)
	≥40	6 (4.51)
Gravity of maternal		
	1	55 (41.35)
	2	58 (43.61)
	≥3	20 (15.04)
BMI (kg/m^2^)		
	Underweight (<18.5)	3 (2.26)
	Normal weight (18.5–24.9)	85 (63.91)
	Overweight (25.0–29.9)	40 (30.08)
	Obese (≥30.0)	5 (3.76)
GA		
	Early (12 weeks)	47 (35.34)
	Mid (22 weeks)	54 (40.60)
	Late (31 weeks)	33 (24.81)
Total		133 (100)

BMI, body mass index; GA, gestational age.

**Table 2:** Intra-operator reproducibility of measurement of fetal cardiac intervals in left ventricle using manual and automated methods.

Variable	Manual Measurement		Automated Measurement	
	ICC (2,1)	95% CI	ICC (2,1)	95% CI
IVCT	0.89	0.85–0.92	0.98	0.97–0.99
IVRT	0.91	0.89–0.94	0.98	0.97–0.99
ET	0.84	0.77–0.88	0.97	0.96–0.98
FT	0.9	0.89–0.95	0.96	0.94–0.97
TI	0.91	0.87–0.94	0.98	0.97–0.99
KI	0.91	0.85–0.94	0.98	0.98–0.99

ICC, intra-class coefficient; CI, confidence interval; IVCT, isovolumetric contraction time; IVRT, isovolumic relaxation time; ET, ejection time; FT, filling time; TI, Tei index; KI, K-index.

## Data Availability

The data are not publicly available due to privacy.

## References

[1] Tei C., Dujardin K.S., Hodge D.O., Kyle R.A., Tajik A.J., Seward J.B. (1996). Doppler index combining systolic and diastolic myocardial performance: Clinical value in cardiac amyloidosis. J. Am. Coll. Cardiol..

[2] Tei C., Ling L.H., Hodge D.O., Bailey K.R., Oh J.K., Rodeheffer R.J., Tajik A.J., Seward J.B. (1995). New index of combined systolic and diastolic myocardial performance: A simple and reproducible measure of cardiac function—A study in normals and dilated cardiomyopathy. J. Cardiol..

[3] Friedman D., Buyon J., Kim M., Glickstein J.S. (2003). Fetal cardiac function assessed by Doppler myocardial performance index (Tei Index). Ultrasound Obstet. Gynecol..

[4] Hernandez-Andrade E., López-Tenorio J., Figueroa-Diesel H., Sanin-Blair J., Carreras E., Cabero L., Gratacos E.A. (2005). Modified myocardial performance (Tei) index based on the use of valve clicks improves reproducibility of fetal left cardiac function assessment. Ultrasound Obstet. Gynecol..

[5] Ichizuka K., Matsuoka R., Hasegawa J., Shirato N., Jimbo M., Otsuki K., Sekizawa A., Farina A., Okai T. (2005). The Tei index for evaluation of fetal myocardial performance in sick fetuses. Early Hum. Dev..

[6] Cruz-Martinez R., Figueras F., Benavides-Serralde A., Crispi F., Hernandez-Andrade E., Gratacos E. (2011). Sequence of changes in myocardial performance index in relation to aortic isthmus and ductus venosus Doppler in fetuses with early-onset intrauterine growth restriction. Ultrasound Obstet. Gynecol..

[7] Figueroa H., Silva M.C., Kottmann C., Viguera S., Valenzuela I., Hernandez A.E., Gratacos E., Antonio A., Illanes S.E. (2012). Fetal evaluation of the modified-myocardial performance index in pregnancies complicated by diabetes. Prenat. Diagn..

[8] Meriki N., Izurieta A., Welsh A.W. (2011). Fetal left modified myocardial performance index: Technical refinements in obtaining pulsed-Doppler waveforms. Ultrasound Obstet. Gynecol..

[9] Van Mieghem T., Gucciardo L., Lewi P., Lewi L., Van Schoubroeck D., Devlieger R., De Catte L., Verhaeghe J., Deprest J. (2009). Validation of the fetal myocardial performance index in the second and third trimesters of gestation. Ultrasound Obstet. Gynecol..

[10] Khandoker A., Kimura Y., Palaniswami M. Automated identification of abnormal fetuses using fetal ECG and doppler ultrasound signals. Proceedings of the 2009 36th Annual Computers in Cardiology Conference (CinC).

[11] Lee M.-Y., Won H.-S., Jeon E.-J., Yoon H.C., Choi J.Y., Hong S.J., Kim M.-J. (2014). Feasibility of using Auto Mod-MPI system, a novel technique for automated measurement of fetal modified myocardial performance index. Ultrasound Obstet. Gynecol..

[12] Ahsan H.K., Marzbanrad F., Kimura T., Saeed A.N., Palaniswami M. Assessing the development of fetal myocardial function by a novel Doppler myocardial performance index. Proceedings of the 2016 38th Annual International Conference of the IEEE Engineering in Medicine and Biology Society (EMBC).

[13] Kozak-Barany A., Jokinen E., Saraste M., Tuominen J., Valimaki I. (2001). Development of left ventricular systolic and diastolic function in preterm infants during the first month of life: A prospective follow-up study. J. Pediatr..

[14] Kozak-Barany A., Jokinen E., Rantonen T., Sarate M., Tuominen J., Jalonen J., Valimaki I. (2000). Efficiency of left ventricular diastolic function increases in healthy full-term infants during the first months of life: A prospective follow-up study. Early Hum. Dev..

[15] Harada K., Takahashi Y., Tamura M., Orino T., Takada G. (1999). Serial echocardiographic and Doppler evaluation of left ventricular systolic performance and diastolic filling in premature infants. Early Hum. Dev..

[16] Scholkmann F., Boss J., Wolf M. (2012). An efficient algorithm for automatic peak detection in noisy periodic and quasi-periodic signals. Algorithms.

[17] Tsutsumi T., Ishii M., Eto G., Hota M., Kato H. (1999). Serial evaluation for myocardial performance in fetuses and neonates using a new Doppler index. Pediatr. Int..

[18] Meriki N., Welsh A.W. (2012). Development of Australian reference ranges for the left fetal modified myocardial performance index and the influence of caliper location on time interval measurement. Fetal Diagn. Ther..

[19] Cruz-Martíne R., Figueras F., Bennasar M., García-Posadas R., Crispi F., Hernández-Andrade E., Gratacos E. (2012). Normal Reference Ranges from 11 to 41 Weeks’ Gestation of Fetal Left Modified Myocardial Performance Index by Conventional Doppler with the Use of Stringent Criteria for Delimitation of the Time Periods. Fetal Diagn. Ther..

